# The clinic application of mNGS and ENA-78 assays to identify intra-amniotic infection/inflammation

**DOI:** 10.3389/fcimb.2025.1510671

**Published:** 2025-04-04

**Authors:** Di Shen, Hui Ju, Hongying Wang, Xietong Wang, Guangzhen Li

**Affiliations:** ^1^ Department of Obstetrics and Gynaecology, Key Laboratory of Birth Regulation and Control Technology of National Health Commission of China, Shandong Provincial Maternal and Child Health Care Hospital Affiliated to Qingdao University, Jinan, China; ^2^ Department of Obstetrics and Gynecology, Liao Cheng People’s Hospital, Liaocheng, China; ^3^ Department of Obstetrics and Gynaecology, Shandong Provincial Hospital Affiliated to Shandong First Medical University, Jinan, China; ^4^ Department of General Surgery, Qilu Hospital of Shandong University, Jinan, China

**Keywords:** preterm labor, intra-amniotic infection/inflammation, metagenomic next-generation sequencing, neutrophil extracellular traps, cervical insufficiency

## Abstract

**Objective:**

The objective of this study is to explore whether metagenomic next-generation sequencing (mNGS) and Epithelial Neutrophil Activating Peptide-78 (ENA-78) assays in the amniotic fluid (AF) of patients with preterm labor (PTL) could be employed for diagnosing intra-amniotic infection/inflammation (IAI/I) and predict the outcomes of emergency cerclage in women with cervical insufficiency(CI).

**Methods:**

AF samples from 40 patients were subjected to PTL were subjected to mNGS and microbial culture to diagnose intra-amniotic infection known as microbial invasion of the amniotic cavity (MIAC); ELISA was used to analyze ENA-78 levels for prediction of intra-amniotic inflammation (IAI). Pregnancy outcomes were compared, the predictive performance of mNGS and ENA-78 were assessed to evaluate the efficacy of emergency cervical cerclage.

**Results:**

The diagnosis rate of MIAC was higher with mNGS (17.5%) compared to microbial culture (2.5%). AF ENA-78 levels were significantly higher in IAI patients than in non-IAI/I patients. ENA-78 demonstrated certain accuracy in identifying IAI, with sensitivity and specificity of 73.3% and 100%, respectively. Compared with non-IAI/I patients, patients with MIAC or IAI exhibited poor pregnancy outcomes after cervical cerclage.

**Conclusions:**

mNGS and ENA-78 assays are valuable means for assessing the state of infection/inflammation in the amniotic cavity and predicting the outcomes of emergency cerclage.

## 1 Introduction

Preterm labor (PTL) represents the primary cause of neonatal death and morbidity globally (Goldenberg et al., 2008). Intra-amniotic infection/inflammation (IAI/I) is a major risk factor for spontaneous PTL. The clinical diagnosis of IAI/I remains challenging. This study intends to study new method and indicator for diagnosing/predicting IAI/I in order to accurately evaluate the amniotic cavity environment.

IAI/I includes both microorganism-positive intra-amniotic infection known as microbial invasion of the amniotic cavity (MIAC), and microorganism-negative intra-amniotic inflammation(IAI). Microbiological research have indicated that 25–40% of PTL patients suffer from MIAC (Onderdonk et al., 2008). However, rapid and accurate identification and diagnosis of intra-amniotic infection poses a significant clinical challenge. Conventional microbial culture possesses several defects including low sensitivity, time-consuming, and limited diagnostic accuracy, resulting in delayed or missed diagnoses. In this context, obstetricians urgently seek a more comprehensive, accurate, and rapid diagnostic methodology. Metagenomic next-generation sequencing (mNGS) can determine pathogenic microorganisms rapidly and accurately by analyzing the content and abundance of DNA and RNA of microorganisms in clinical samples, which is used for diagnosis of infectious diseases.

To date, mNGS approaches have been successfully applied to various samples such as blood, respiratory secretion, cerebrospinal fluid, fecal, and urine. Nevertheless, there has been no previous report on the application of mNGS for detecting microorganisms in amniotic fluid (AF).

In PTL patients with intact membranes, IAI is more prevalent than MIAC (Gilman-Sachs et al., 2018). The majority of IAI patients exhibit no clinical symptoms of inflammation, hindering timely diagnoses (Cobo et al., 2018). Therefore, in addition to improving the diagnostic rate of MIAC, it is critical to identify inflammation indicators with high sensitivity and specificity to facilitate early diagnosis of IAI. Therefore, in addition to improving the diagnosis rate of MIAC, identifying inflammatory indicators with high sensitivity and specificity is also crucial for the early diagnosis of IAI.

Many studies in this field have focused on classic inflammatory markers, such as cytokines (IL-1, IL-6, IL-8 and TNF-a) and MMPs (MMP-8 and MMP-9) (Georgiou et al., 2015). Neutrophil extracellular traps (NETs), emerging as new biomarkers of infection and inflammation, are extracellular reticular structures composed of a DNA skeleton and a variety of granule proteins such as myeloperoxidase (MPO) and neutrophil elastase (NE). released after neutrophils activation (Barnado et al., 2016; Martinez-Varea et al., 2017). Our previous studies have found that NETs contribute to PTL by inducing apoptosis of amniotic epithelial cells (Hu et al., 2023). Epithelial Neutrophil Activating Peptide-78 (ENA-78) is responsible for the recruitment and activation of neutrophils (Schnyder-Candrian and Walz, 1997), involved in the inflammatory pathological process of diseases. ENA-78, activates NADPH oxidase in neutrophils to produce ROS, and the activation of NADPH/ROS pathway is a crucial step in NETs release, potentially related to NETs generation, making it a promising new marker for predicting IAI.

PTL is typically unavoidable when a patient presents with cervical dilatation and protrusion of the fetal membranes due to cervical insufficiency (CI). Emergency cervical cerclage is an effective therapy for CI to prevent PTL (Friedman and Cleary, 2014). Prior studies had indicated that intra-amniotic infection/inflammation (IAI/I) presented in 13–51% of CI patients with bulging membranes, with IAI/I being the primary determinant of cervical cerclage efficacy. Thus, the rapid and accurate identification of IAI/I is of great significance in guiding clinical treatment and management of CI.

In conclusion, the primary objective of this study is to evaluate the utility of mNGS and ENA-78 in the identification of IAI/I and the guiding significance in the clinical treatment of CI.

## 2 Materials and methods

### 2.1 Patients

The retrospective cohort enrolled 40 PTL patients with intact fetal membranes who were admitted to Shandong Maternal and Child Health Hospital Affiliated to Qingdao University in 2018-2022. All patients had signed written Informed Consent. The results of prenatal screening or prenatal diagnosis were negative for all patients. Thirty-eight of the patients diagnosed with CI, presented with progressive painless cervical dilation and fetal membrane bulge, these patients underwent emergency cervical cerclage. The study design was approved by the Ethics Committee of Shandong Maternal and Child Health Hospital Affiliated to Qingdao University (NO.2024-018) and was conducted in accordance with the guidelines of the 1964 Declaration of Helsinki in March 4, 2024.

### 2.2 Amniotic fluid microbial culture

AF was extracted with ultrasound-guided amniocentesis. Specifically, a puncture needle was used to penetrate the abdominal wall and myometrium into the amniotic cavity, from which 30 ml of AF was extracted with a syringe. One third of the resulting sample was cultured with standard microbial culturing methods immediately the remaining 20 ml were analyzed with mNGS and enzyme-linked immunosorbent assay (ELISA).

### 2.3 mNGS

To ensure that all potential pathogens were detected in the AF samples, both DNA and RNA were extracted for sequencing, mNGS sequencing, raw data analysis, bioinformatics analysis were conducted by Yinfeng Gene Technology Co. Ltd and Willingmed Medical Technology Co., Ltd.

### 2.4 MMP-8 and ENA-78 detection by ELISA

AF was centrifuged and the supernatant stored at -80°C until further analysis. MMP-8 and ENA-78 levels were measured with an ELISA kit (Boster, Wuhan, China). IAI was determined setting a concentration threshold of 23ng/ml for MMP-8.

### 2.5 Quantitative detection of NETs

Cell free-DNA (cf-DNA) was quantified in the AF samples using the Quant-It™ PicoGreen™ dsDNA Test Kit (Invitrogen, Carlsbad, CA, USA). AF levels of NE and MPO were measured using an ELISA kit (Boster, Wuhan, China).

### 2.6 Live-cell imaging and multiplex immunofluorescence

#### 2.6.1 Live-cell imaging

Human neutrophils (3×10^4^ cells/ml) were plated on 96-well plates coated with poly-L-lysine (Solarbio,Beijing,China) and incubated with PMA (MedChemExpress, New Jersey, USA) or ENA-78 (MedChemExpress, New Jersey, USA), or leave untreated for 3 hours at 37 ^°^C in a 5% CO_2_ atmosphere. NETs were detected using a mixture of cell-permeable (Hoechst 33342; Solarbio,Beijing,China) and cell-impermeable (Sytox Green; Invitrogen Carlsbad, CA,USA) fluorescent DNA dyes. The proportion of neutrophils forming NETs (NET%) was calculated as follows: (number of cells showing NETs/total number of cells) × 100%.

#### 2.6.2 Multiplex immunofluorescence

Inoculate neutrophils (1×10^5^ cells/mL) into a 24 well plate with coverslips coated with poly-L-lysine) (Solarbio, Beijing, China), and then cultivated with PMA or ENA-78, or leave untreated for 3 hours. Then, the cells were fixed in 4% paraformaldehyde, impermeable, and sealed with goat serum (Boster, Wuhan, China) at 37°C for 30 minutes. The anti-MPO (rabbit, 1:100, Abcam, Cambridge, UK) and anti-NE (mouse, 1:100, Abcam, Cambridge, UK) primary antibodies were added to coverslips, overnight at 4°C, and then the second antibody Rabbit-FITC (Boster, Wuhan, China) and murine-CY3 (Boster, Wuhan, China) were added and treated at 37°C for 30 minutes. DAPI (Solarbio, Beijing, China) is used to detect DNA. The slides were observed by fluorescence microscope (Olympus, Tokyo, Japan).

### 2.7 Emergency cervical cerclage

Emergency cervical cerclage was performed in CI patients with bulging membranes. If the pregnancy was successful, cerclages were removed at 36 weeks of gestation and vaginal delivery was encouraged unless obstetric factors required cesarean section. Pregnancy outcome was determined by comparing these indicators (amniocentesis-to-delivery interval, abortion rate<28 weeks of gestation, delivery rate<37weeks of gestation, mean NICU referral rate,mean neonatal survival rate, mean the average birth weight) among the groups.

### 2.8 Statistical analysis

The mean and standard error of the mean (SEM) were calculated for all analyzed parameters. Data analyses were performed in GraphPad Prism 9.0 (GraphPad Software Inc., La Jolla, CA, USA). Comparisons between treatment groups were assessed with Student’s *t*-test for pairwise comparisons or Rank sum test(ANOVA). Differences were considered statistically significantly different at *p* < 0.05. *A* receiver operating characteristic (ROC) curve was used to evaluate the diagnostic performance of ENA-78 for IAI.

## 3 Results

### 3.1 Pathogens detected by microbial culture and mNGS

**Figure 1:**
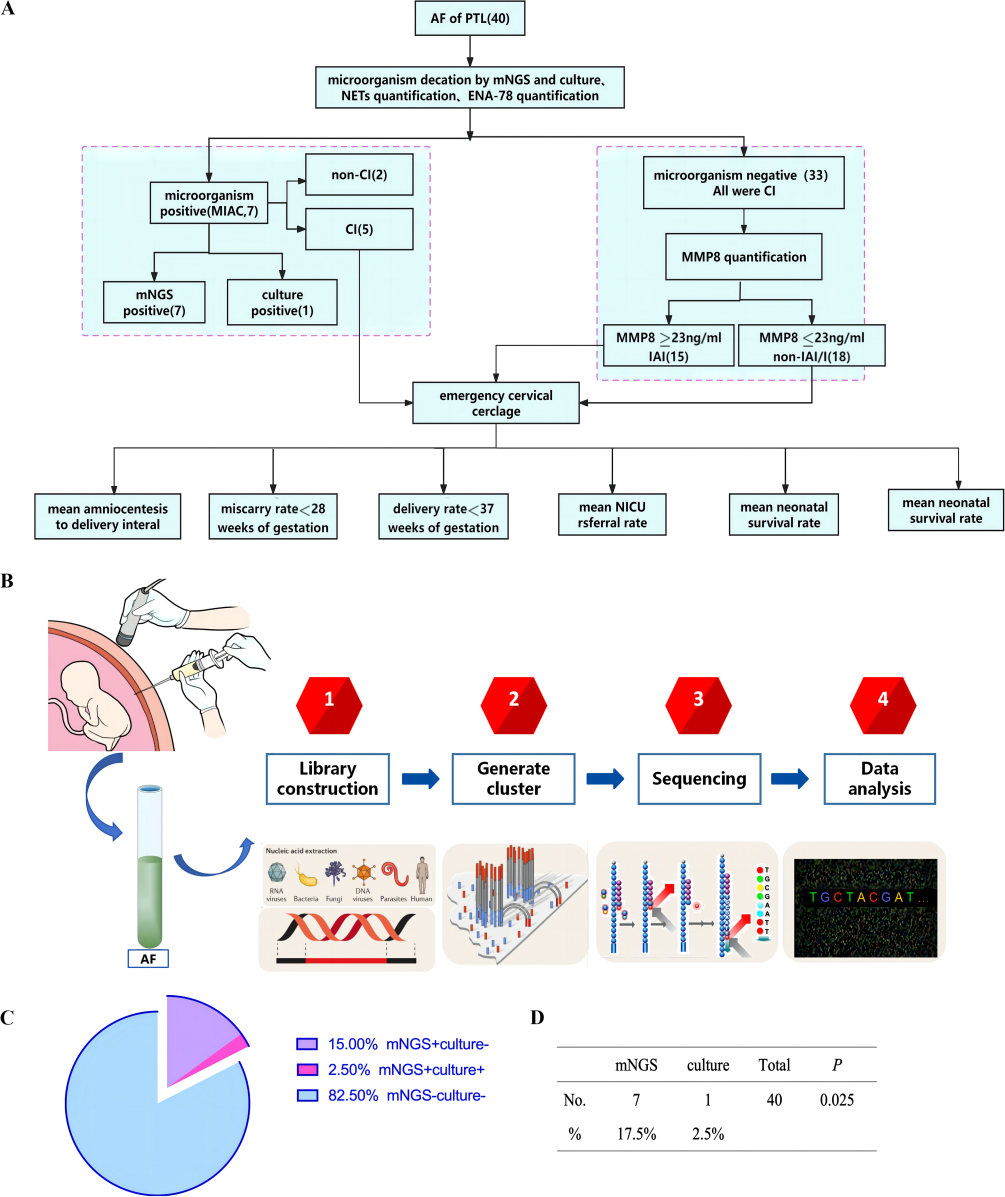
**(A)** The work flow of study design. **(B)** The process of detecting microorganisms in AF by mNGS. **(C)** The detection rate of the mNGS and culture. The detection rate of mNGS was higher than that of culture, *p*<0.05. **(D)** AF samples from 40 PTL patients were analyzed for the presence of pathogens using both mNGS and microbial culture. Based on microbial culture solely, 2.5% (1/40) of the patients identified with MIAC. mNGS detected microorganisms in 17.5% (7/40) of the patients, *p*<0.05. AF, amniotic fluid; CI, cervical insufficiency; PTL, Preterm labor; MIAC, microbial invasion of the amniotic cavity; IAI, intra-amniotic inflammation; non-IAI/I, non-intra-amniotic infection/inflammation; NICU, neonatal intensive care unit; mNGS, Metagenomic next-generation sequencing.

AF samples from 40 PTL patients were analyzed for the presence of pathogens using both mNGS and microbial culture. Based on microbial culture solely, 2.5% (1/40) of the patients identified with MIAC. However, mNGS detected microorganisms in 17.5% (7/40) of the patients, representing a significantly higher positive rate compared to microbial culture (*p*<0.05) (**Figure 1** and **Table 1**). Notably, three microorganism-positive samples detected with mNGS were determined to be mixed infections. The MIAC case detected with microbial culture was classified with that method only as an anaerobic bacterial infection, whereas mNGS analysis clearly identified the presence of both Bacteroides fragilis and Campylobacter ureolyticus in the sample (**Table 1**). Thus, mNGS not only significantly improved the microorganism detection rate in AF samples, but also directly identified the bacterial species present. This highlights the great advantages of mNGS in patients with mixed infections.

**Table 1:** Pathogenic microorganism detected by mNGS and microbial culture.

Method	Microorganisms	Case	Positive rate
mNGS	Ureaplasma parvo	2	17.5%
	Bacteroides fragilis Campylobacter ureolyticus	1	
	Aerococcus christensenii	1	
	Streptococcus anginosus	1	
	Fusobacterium nucleatum Alloscardovia omnicolens	1	
	Klebsiella pneumoniae Enterococcus faecalis	1	
microbial culture	Anaerobe	1	2.5%

mNGS, Metagenomic next-generation sequencing.

### 3.2 Predictive performance of ENA-78

**Figure 2:**
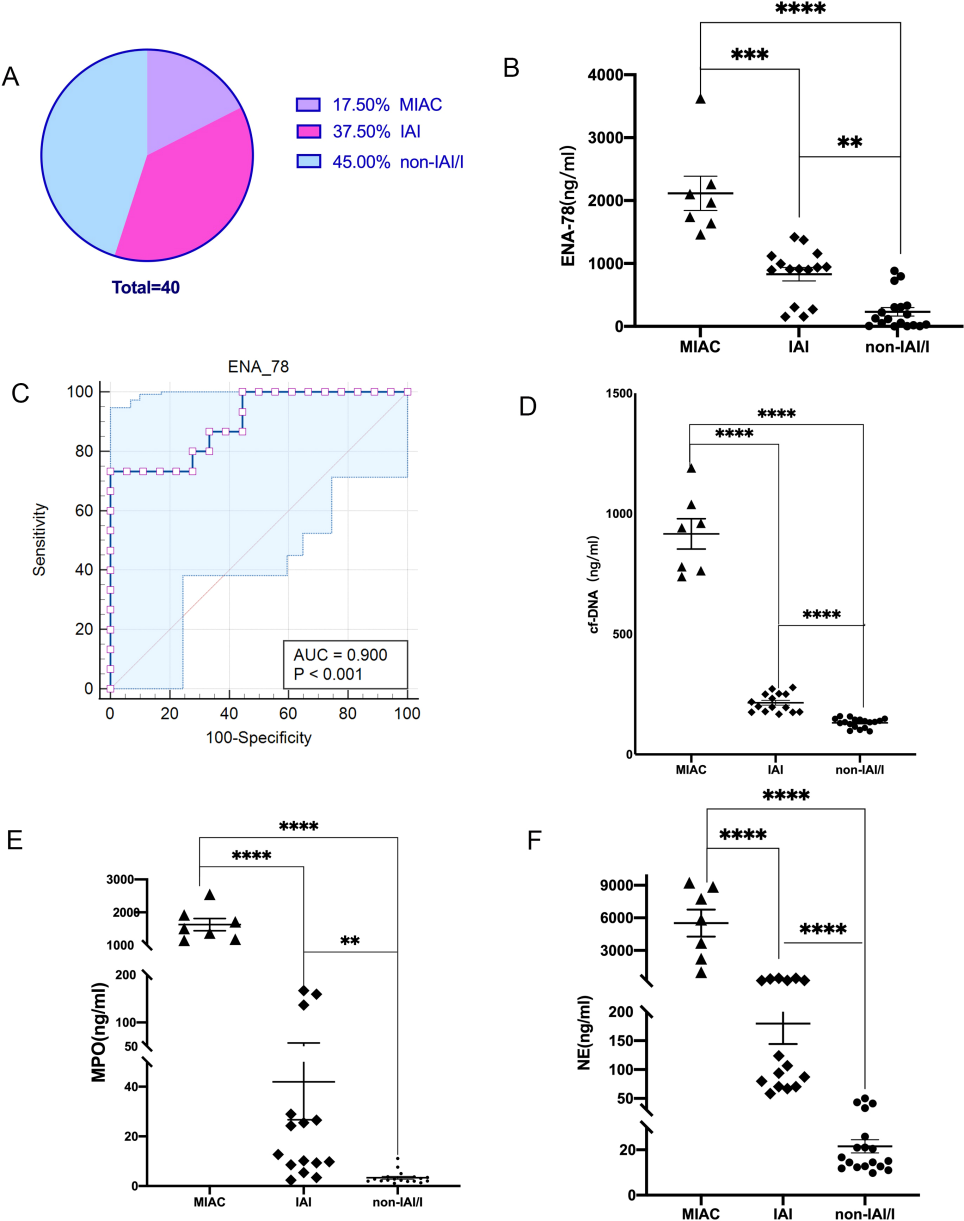
**(A)** Among the 40 preterm patients, the incidence of IAI was significantly higher than MIAC. **(B)** The ENA-78 concentration in AF of MIAC and IAI were higher than that of non-IAI/I, **p<0.01, **** *p*<0.0001. **(C)** The AUC of ENA-78 is 0.900, and the sensitivity and specificity was 73.3% and 100% respectively. **(D-F)** The NETs-makers concentration of AF was highest in MIAC group, middle in IAI group, and lowest in non-IAI/I group, ****p<0.0001. Data are presented as the mean ± SEM. Comparisons between the different groups were performed using Student’s t-test. MIAC, microbial invasion of the amniotic cavity; IAI, intra-amniotic inflammation; non-IAI/I, non-intra-amniotic infection/inflammation; ENA-78, Epithelial Neutrophil Activating Peptide-78; AUC, Area Under Curve; NETs, Neutrophil extracellular traps; cf-DNA, cell free-DNA; MPO, Myeloperoxidase; NE, neutrophil elastase; SEM, standard error of the mean.

MIAC are defined as the detection of any microorganisms by mNGS or microbial culture, while IAI refer to AF MMP-8 concentration ≥ 23ng/ml in the case of microorganisms negative. Among the 40 PTL patients, there were 7(17.5%) cases of MIAC (Group1) and 15 (37.5%) cases of IAI(Group2). The remaining 18(45%) cases were classified as non-IAI/I(Group3). ENA-78 levels were higher in patients of MIAC(2116 ± 272.4ng/ml) and IAI(829.4 ± 107.0ng/ml)) compared to non-IAI/I(232.0 ± 67.34ng/ml) group, with statistically significant. We calculated the receiver operating characteristics curve for predicting IAI. Area under the ROC curve(AUC) is 0.900. ENA78 demonstrated certain accuracy in diagnosing IAI, with the sensitivity and specificity was 73.3% and 100% respectively (**Figure 2**).

### 3.3 The concentration of NETs

The AF of MIAC、;IAI、;non-IAI/I patients was examined to evaluate the levels of NETs. The level of NETs-makers: cf-DNA, MPO, NE in MIAC and IAI patients were higher than those in non-IAI/I patients(915.8 ± 62.87 vs214.4 ± 9.953 vs 131.0 ± 4.604ng/ml,1628 ± 185.8vs41.95 ± 15.23vs3.342 ± 0.5791ng/ml,5519 ± 1240vs179.6 ± 35.27vs21.51 ± 2.902ng/m. There was a statistical difference in all the markers between any two groups (**Figures 2D–F**). It is evident that the NETs levels in IAI patients were significantly higher than those in non-IAI/I patients.

### 3.4 ENA-78 promotes NETs generation

**Figure 3:**
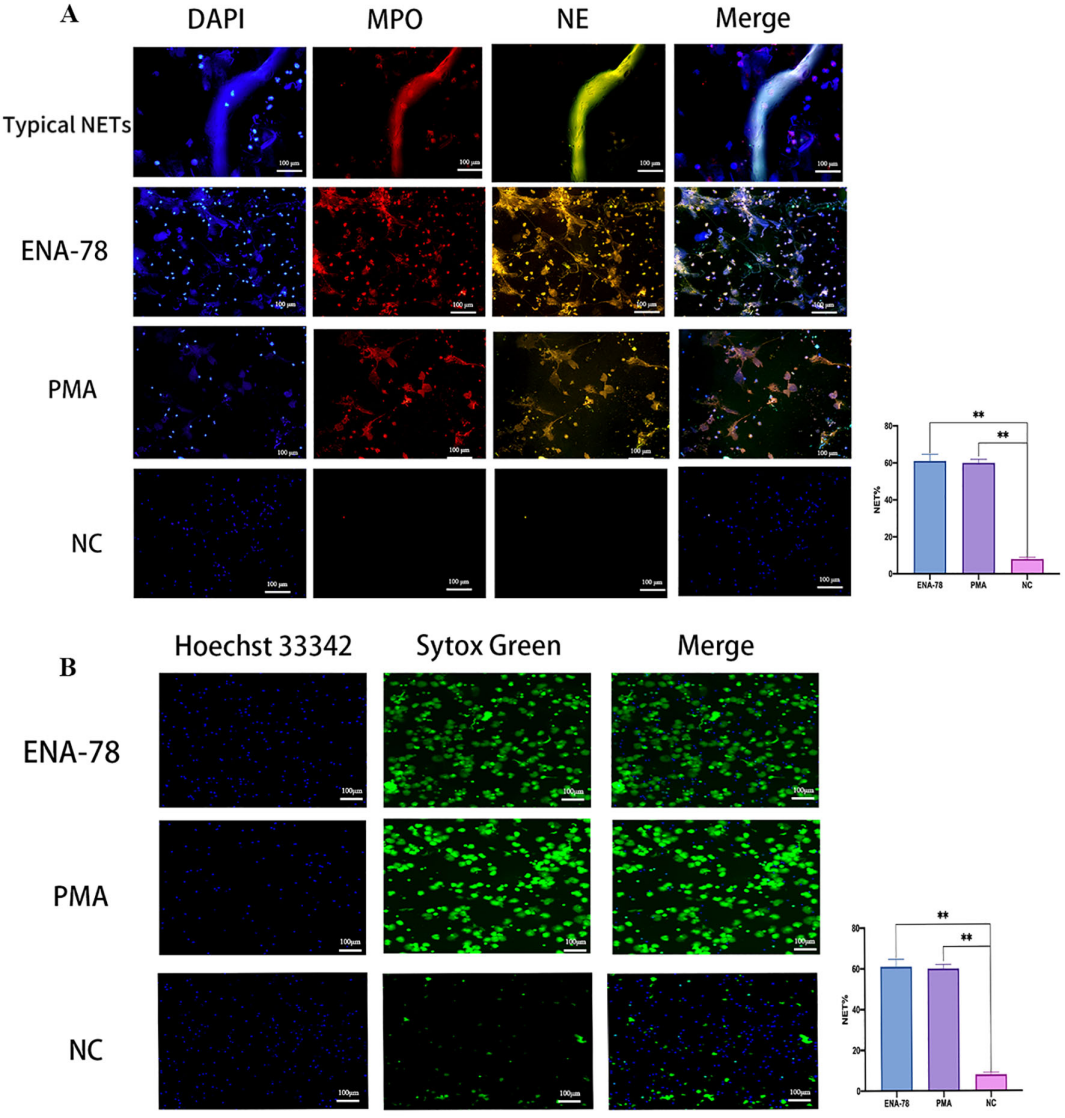
ENA-78 promotes NETs generation. **(A)** Typical NETs structures were co-localized with MPO (red), NE (green), and DNA(blue). NETs production after ENA-78, PMA stimulation were significantly higher than NC, original magnification x 20, scale bars = 100μm. **(B)** Live-cell imaging shows NETs skeleton: cf-DNA, Sytox Green staining represents extracellular DNA; Hoechst 33342 staining represents intracellular DNA, original magnification x 10, Scale bars =100μm, **P < 0.01; Data are presented as the mean ± SEM. Comparisons between the different groups were performed using Student’s t-test or one-way ANOVA. ENA-78, Epithelial Neutrophil Activating Peptide-78; NETs, Neutrophil extracellular traps; cf-DNA, cell free-DNA; MPO, Myeloperoxidase; NE, neutrophil elastase; DAPI:4’,6-diamidino-2-phenylindole; PMA, phorbol-12-myristate-13-acetate; NC, negative control; SEM, standard error of the mean.

Neutrophils were incubated with ENA-78 to evaluate its positivity effect on NETs release, PMA was used as positive control. Results from Live-cell imaging and mIF showed that the release of NETs was significantly increased after treatment with ENA-78 compared with the negative control group, the difference was statistically significant, suggesting that ENA-78 promoted the generation of NETs (**Figure 3**).

### 3.5 Effects of IAI/I on emergency cervical cerclage efficacy

Out of forty preterm patients, thirty-eight were diagnosed with cervical insufficiency (CI) (**Figure 1**). No significant differences were found in clinical characteristics such as maternal age, gestational age, and cervical dilatation among the three groups (**Table 2**). The amniocentesis - to - delivery interval was the shortest in the MIAC group and the longest in the non - IAI/I group (**Table 3**). Moreover, the MIAC group presented the lowest birth weight (959.8 ± 160.4g) and neonatal survival rate (20%), the highest referral rate to the neonatal NICU (100%), and the highest abortion rate before 28 weeks of gestation (80%), with *p* < 0.05. The neonatal survival rate in both the non - IAI/I and IAI groups was 100%, and the abortion rate before 28 weeks of gestation in both groups was 0. Notably, compared with non - IAI/I patients (3234 ± 92.72g), IAI patients had a lower neonatal birth weight (2303 ± 203.2g). Significantly, the delivery rate before 37 weeks of gestation (66.67% vs 16.67%) and the NICU referral rate (40% vs 5.6%) were higher in IAI patients than in non - IAI/I patients (*p*<0.05). These results indicate that MIAC has the worst pregnancy outcomes, while non - IAI/I has the best. This suggests that the presence of IAI/I has a detrimental impact on the outcome of emergency cervical cerclage. Therefore, the assessment of infection/inflammation in the amniotic cavity through mNGS and ENA - 78 may have predictive value for the pregnancy outcomes of patients with CI who undergo emergency cervical cerclage.

**Table 2:** Clinical characteristics of patients undergone emergency cervical cerclage.

	MIAC G1 (n=5)	IAI G2 (n=15)	non-IAI/I G3 (n=18)	G1vsG2	*P* G1vsG3	G2vsG3
Maternal age (y)	31.4 ± 0.8718	31.73 ± 1.213	32.67 ± 0.9967	ns	ns	ns
GA at sampling (w)	24.28 ± 0.4234	24.51 ± 0.4719	23.6 ± 0.4401	ns	ns	ns
Cervical dilatation (cm)	4.2 ± 1.655	3.6 ± 0.7856	4.6 ± 0.8130	ns	ns	ns
primipara	60% (3/5)	60% (9/11)	61% (11/18)	ns	ns	ns
women who had cone biopsies	0 (0/5)	0 (9/11)	0 (11/18)	ns	ns	ns
Use of antibiotics	100% (5/5)	100% (15/15)	100% (18/18)	ns	ns	ns

Data are presented as the mean ± SEM. ns, not significant; G, group; MIAC, microbial invasion of the amniotic cavity; IAI, intra-amniotic inflammation; non-IAI/I, intra-amniotic infection/inflammation; GA, gestational age.

**Table 3:** Comparison of pregnancy outcomes between patients.

	MIAC	IAI	non-IAI/I		*P*	
	G1(n=5)	G2(n=15)	G3(n=18)	G1vsG2	G1vsG3	G2vsG3
ENA-78(pg/ml)	2116 ± 272.4	829.4 ± 107.0	232.0 ± 67.34	<0.0001	<0.001	<0.0001
cf-DNA(ng/ml)	915.8 ± 62.87	214.4 ± 9.953	131.0 ± 4.604	<0.0001	<0.0001	<0.0001
MPO(ng/ml)	1628 ± 185.8	41.95 ± 15.23	3.342 ± 0.5791	<0.0001	<0.0001	0.091
NE(ng/ml)	5519 ± 1240	179.6 ± 35.27	21.51 ± 2.902	<0.0001	<0.0001	<0.001
Mean amniocentesis-to-delivery interval(d)	10.6 ± 5.066	68.7 ± 5.634	105.6 ± 2.48	<0.0001	<0.0001	<0.0001
Abortion rate<28 weeks of gestation(%)	80%(4/5)	0(0/15)	0(0/18)	0.001	<0.0006	ns
Delivery rate<37weeks of gestation(%)	100%(5/5)	66.67%(6/15)	16.67%(3/18)	0.003	0.0017	0.2395
Mean NICU referral rate(%)	100%(5/5)	40%(6/15)	5.6%(1/18)	0.0379	0.0002	0.03
Mean neonatal surviva lrate(%)	20%(1/5)	100%(15/15)	100%(18/18)	0.001	0.0006	ns
Mean the average birth weight(g)	959.8 ± 160.4	2303 ± 203.2	3234 ± 92.72	0.0019	<0.0001	0.0081

Data are presented as the mean ± SEM. Significant: *p*<0.05. ns, not significant; G, group; MIAC, microbial invasion of the amniotic cavity; IAI, intra-amniotic inflammation; non-IAI/I, intra-amniotic infection/inflammation; GA, gestational age; ENA-78, Epithelial Neutrophil Activating Peptide-78; NETs, Neutrophil extracellular traps; cf-DNA, cell free-DNA; MPO, Myeloperoxidase; NE, neutrophil elastase.

## 4 Discussion

### 4.1 Diagnostic value of mNGS for MIAC

MIAC is closely associated with PTL (da Fonseca et al., 2020). However, the detection rate might be underestimated due to the low sensitivity of conventional microbial culture (Romero et al., 2023). Different from traditional culture-based diagnostics, mNGS can detects over 10,000 pathogens by extracting nucleic acids from amniotic fluid. This approach has unique significance in diagnosing infections that are difficult to detect and insufficiently studied, as well as in uncovering novel and emerging pathogens (Goldberg et al., 2015; Miao et al., 2018). The most prevalent microorganisms of MIAC is *Mycoplasma urealyticum*, and *Fusobacterium nucleatum* is also among the most frequently detected microbial species in the AF of PTL patients (Romero et al., 2023; Gomez-Lopez et al., 2022). Other commonly encountered microorganisms in AF include *Listeria monocytogenes*, *Mycoplasma hominis*, *Streptococcus agalactiae*, *Escherichia coli*, *Fusobacterium* spp. and *Gardnerella vaginalis (* Mendz et al., 2013). In this study, a total of 40 AF samples underwent both mNGS and microbial culture. Microbial culture revealed the presence of microorganisms in only one AF sample and failed to determine the strain identity. In contrast, mNGS identified nine microbial species in seven AF samples: *Ureaplasma parvus*, *Aerococcus kiri*, *Streptococcus angina*, *Bacteroides tenuis* complicated with *Campylobacter urealyticus*, *Clostridium nucleatum* complicated by *Isoscadovitis universalis*, and *Klebsiella pneumoniae* complicated by *Enterococcus faecalis*. These results were generally consistent with previous reports on microbes identified in AF samples. Notably, mNGS indicated that 42.8% of microorganism-positive patients were co-infected with two species of bacteria; while microbial culture detected only a single kind of pathogen in one positive case, and had a lower microorganism-positive rate. Significantly, the mNGS results were obtained on the second day of specimen inspection. This shorter time span compared with microbial culture (3-5days) significantly enhanced the diagnostic efficiency. Consequently, our experiments demonstrated that using mNGS for MIAC diagnoses can improve the pathogen detection rate, reduce the detection time, and enable the simultaneous detection of multiple pathogens. Furthermore, this approach can be cautiously applied to detect pathogens that cannot be identified by other existing techniques.

Although our results clearly showed that mNGS outperformed culture-based methods, popularization of this approach confronts several challenges. In China, mNGS assays of a single sample costs $500–600, which hinders its clinical implementation. Moreover, standardized operating procedures, universal reference standards, quality control, and interpretation of mNGS data lack expert consensus in clinical laboratories.

Despite these limitations,with the continuous development and maturation of relevant technologies, mNGS is anticipated to function as an effective complement to traditional methods.

### 4.2 Diagnostic value of ENA-78 for IAI

Delivery commences with the uterus transitioning from a resting state to a contractile one. This transition may occur partially due to alterations in cellular signaling from the anti-inflammatory to the pro-inflammatory pathway (Conde-Agudelo and Romero, 2014; Romero et al., 2014a). A multitude of studies and resources have been devoted to identifying biomarkers corresponding to this shift from an anti-inflammatory to a pro-inflammatory response. The aim is to determine markers that can accurately predict PTL (Manokhina et al., 2017).

Previous studies have shown that IAI may also contribute to PTL (Romero et al., 2014a; Šket et al., 2021). Indeed, the incidence of IAI has been reported to be significantly higher than that of MIAC among PLT patients (Romero et al., 2014b). Consistent with those findings, our current study revealed that 45% of PTL patients suffered from IAI, while only 17.5% were afflicted with MIAC. Timely diagnosis of IAI is particular important for the treatment of PTL. Therefore, exploring accurate, sensitive, specific predictive indicators of IAI is crucial. Although some inflammatory markers (e.g., MMP8 and IL-6) have been reported as predictors of IAI (Liu et al., 2016; Leaños-Miranda et al., 2021), the insufficient clinical accessibility of assays for these markers means they have not been widely used in clinical practice. Thus, the present research focused on evaluating whether ENA-78 detection in PTL patients can assist in predicting IAI, with the intention of guiding clinical treatment.

AF ENA-78 is produced by amnion and chorion and play a role in boht normal and pathological pregnancy (Laudanski et al., 2014). ENA-78 levels were remarkably elevated in AF of MIAC patients. This elevation is responsible for leukocytosis in the foetal membrane, resulting in inflammatory activation, matrix remodeling, membrane rupture, and initiation of uterine contractions. So, ENA-78 play a role in the mechanism of infection-driven PTL and membranes rupture secondary to neutrophil recruitment and activation. We also observed elevated levels of ENA-78 in AF of IAI patients, the AUC of ENA-78 is 0.900, and the sensitivity and specificity was 73.3% and 100% respectively, suggesting that ENA-78 has certain diagnostic performance for IAI.

ENA-78 is a potent chemoattractant and activator of neutrophils, and its level of is positively correlated with the degree of neutrophils infiltration. The increased neutrophils at the maternal fetal interface participate in the pathological process of PTL induced by IAI/I. Therefore, we measured NETs levels in AF because NETs are one of the main functional forms of neutrophils. Notably, we also found that NETs levels were significantly increased in the AF of MIAC and IAI patients compared with non-IAI patients, with the highest level of NETs observed in MIAC patients.

Our previous research indicated that NETs infiltrate extensively in the amniotic membranes of PTL patients, contributing to PTL by promoting hAECs apoptosis and the degradation of amniotic ECM (Hu et al., 2023). In MIAC patients, neutrophils are recruited to the AF to entrap pathogens and defend against infection by releasing NETs (Gomez-Lopez et al., 2017b; Gomez-Lopez et al., 2017a; Driouich et al., 2019). Among microorganism-negative IAI patients, alarmins (Tadie et al., 2013), heme (Nader et al., 2020), and cytokines (e.g., IL-1 and IL-8) (Brinkmann et al., 2004) may also induce NETs formation. ENA-78, a kind of cytokine, activates NADPH oxidase in neutrophils, which plays a key role in the production of NETs. So the increase of NETs in AF of IAI patients may be closely related to ENA-78. ENA-78 may be involved in PTL by regulating NETs generation. In this study, we confirmed that ENA-78 can induce NETs release by live cell fluorescent staining and multi-cell fluorescence staining *in vitro*. In conclusion, we have reasons to believe that ENA-78 is expected to be a reliable indicator for diagnosing IAI.

However, our research has limitations because our sample size is not large enough to summarize the threshold for diagnosing IAI. Next, we will recruit more patients for in-depth research to further evaluate the expression level of ENA-78 in IAI patients and its role in driving PTL.

### 4.3 Clinical significance of mNGS and ENA-78 in detecting AF

A high rate of IAI/I has been reported in cases of of PTL induced by CI. This indicates that IAI/I is an crucial factor affecting pregnancy outcomes (Mays et al., 2000; Hassan et al., 2001; Lee et al., 2008). Therefore, rapid, accurate identification of IAI/I may guide clinical management and decision making. In the present study, emergency cervical cerclage was performed in 38 PTL patients diagnosed with CI due to cervical dilation and fetal membrane bulge. First, the amniotic cavity environment was evaluated using mNGS and ENA-78 assays to identify IAI/I. Subsequently, the guiding value of the results in the implementation of emergency cervical cerclage was assessed.

Evidence indicates that the pregnancy outcomes of CI patients with IAI/I after emergency cervical cerclage are relatively poor. Overall, 13–51% of patients with CI and bulging fetal membranes have IAI/I (Lee et al., 2008; Oh et al., 2010). Among CI patients presenting in healthcare settings with MIAC, 76% deliver within 48 h of admission (Romero et al., 1992). Patients without IAI/I who undergo emergency cervical cerclage deliver at a significantly later gestational age and have a higher neonatal survival rate than those with IAI/I (Mays et al., 2000). Our results were generally consistent with these earlier findings, 52.6% of patients with CI were diagnosed with IAI/I, with 13.5% and 39.47% having MIAC and IAI, respectively. MIAC patients had the shortest amniocentesis-to-delivery interval, the lowest neonatal survival rate and birth weight. The prognosis was worst among MIAC patients, with four of five newborns dying, the associated amniocentesis-to-delivery intervals were 1, 3, 5, and 16 days. It is noteworthy to highlight the high rate of neonatal mortality in MIAC patients, emergency cervical cerclage did not seem to be beneficial for improving pregnancy outcomes. In view of this, we proposed to performing AF mNGS assays for CI patients in advance to facilitate the pregnancy outcome prediction and guide the clinical treatment.

Compared to non-IAI/I patients, IAI patients exhibited a higher NICU referral rate, a shorter average amniocentesis-to-delivery interval, and a lower the average birth weight. These findings suggest that ENA-78 levels may affect pregnancy outcomes among patients who have undergone emergency cervical cerclage. The pregnancy outcome of IAI patients with higher level of ENA-78 were worse than those of non-IAI/I. However, all newborns delivered after 28 weeks and survived. It can be seen that emergency cervical cerclage is worthy of recommendation for IAI patients with cervical dilatation and protrusion of the fetal membranes. Otherwise, abortion is very likely to occur. Preoperative determination of AF ENA-78 concentration to determine intrauterine environment may have predictive value for the surgical curative effect.

Currently, emergency cervical cerclage is still controversial for the treatment of CI patients complicated with IAI/I (Giouleka et al., 2023), so appropriate parameters for studing and assessing the surgical effects need to further researched. Given the accuracy of AF microorganism detection with mNGS and the apparent predictive value of ENA-78 levels for pregnancy outcome, these techniques may aid in clinical decision-making. We suggest that these methods be widelyy implemented as part of the preoperative evaluation.

In summary, mNGS and ENA-78 assays in AF have compelling application value and clinical significance for the identification of IAI/I. Continuous improvement of these methods will endow them with even more important roles in future clinical practice. Overall, our findings provide specific suggestions for increasing accurate IAI/I diagnosis rates and guiding clinical management of CI, contributing to improved pregnancy outcomes and reduced neonatal mortality.

## Data Availability

The original contributions presented in the study are included in the article/**Supplementary Material**, further inquiries can be directed to the corresponding author/s.

## References

[B1] Barnado A. Crofford L. J. Oates J. C. (2016). At the Bedside: Neutrophil extracellular traps (NETs) as targets for biomarkers and therapies in autoimmune diseases. J. leukocyte Biol. 99, 265–278. doi: 10.1189/jlb.5BT0615-234R 26658004 PMC6608010

[B2] Brinkmann V. Reichard U. Goosmann C. Fauler B. Uhlemann Y. Weiss D. S.. (2004). Neutrophil extracellular traps kill bacteria. science 303, 1532–1535. doi: 10.1126/science.1092385 15001782

[B3] Cobo T. Kacerovsky M. Jacobsson B. (2018). Noninvasive sampling of the intrauterine environment in women with preterm labor and intact membranes. Fetal diagnosis Ther. 43, 241–249. doi: 10.1159/000480232 29080890

[B4] Conde-Agudelo A. Romero R. (2014). Prediction of preterm birth in twin gestations using biophysical and biochemical tests. Am. J. Obstet Gynecol 211, 583–595. doi: 10.1016/j.ajog.2014.07.047 25072736 PMC5898802

[B5] da Fonseca E. B. Damião R. Moreira D. A. (2020). Preterm birth prevention. Best Pract. Res. Clin. Obstetrics Gynaecology 69, 40–49. doi: 10.1111/brv.125220 33039310

[B6] Driouich A. Smith C. Ropitaux M. Chambard M. Moore J. P. (2019). Root extracellular traps versus neutrophil extracellular traps in host defence, a case of functional convergence? Biol. Rev. 94 (5), 1685–1700. doi: 10.1111/brv.125220 31134732

[B7] Friedman A. M. Cleary K. L. (2014). Prediction and prevention of ischemic placental disease. Semin. perinatology; 2014: Elsevier; p, 177–182. doi: 10.1053/j.semperi.2014.03.002 24836830

[B8] Georgiou H. M. Di Quinzio M. K. Permezel M. Brennecke S. P. (2015). Predicting preterm labour: current status and future prospects. Dis. Markers 2015 (9), 1–9. doi: 10.1155/2015/435014 PMC4486247 26160993

[B9] Gilman-Sachs A. Dambaeva S. Garcia M. D. S. Hussein Y. Kwak-Kim J. Beaman K. (2018). Inflammation induced preterm labor and birth. J. Reprod. Immunol. 129, 53–58. doi: 10.1016/j.jri.2018.06.029 30025845

[B10] Giouleka S. Boureka E. Tsakiridis I. Siargkas A. Mamopoulos A. Kalogiannidis I.. (2023). Cervical cerclage: A comprehensive review of major guidelines. Obstetrical Gynecological Survey 78, 544–553. doi: 10.1097/OGX.0000000000001182 37976303

[B11] Goldberg B. Sichtig H. Geyer C. Ledeboer N. Weinstock G. M. (2015). Making the leap from research laboratory to clinic: challenges and opportunities for next-generation sequencing in infectious disease diagnostics. MBio 6, e01888–e01815. doi: 10.1128/mBio.01888-15 26646014 PMC4669390

[B12] Goldenberg R. L. Culhane J. F. Iams J. D. Romero R. (2008). Epidemiology and causes of preterm birth. Lancet 371, 75–84. doi: 10.1016/S0140-6736(08)60074-4 18177778 PMC7134569

[B13] Gomez-Lopez N. Galaz J. Miller D. Farias-Jofre M. Liu Z. Arenas-Hernandez M.. (2022). The immunobiology of preterm labor and birth: intra-amniotic inflammation or breakdown of maternal–fetal homeostasis. Reproduction 164, R11–R45. doi: 10.1530/REP-22-0046 35559791 PMC9233101

[B14] Gomez-Lopez N. Romero R. Garcia-Flores V. Xu Y. Leng Y. Alhousseini A.. (2017a). Amniotic fluid neutrophils can phagocytize bacteria: a mechanism for microbial killing in the amniotic cavity. Am. J. Reprod. Immunol. 78, e12723. doi: 10.1111/aji.2017.78.issue-4 PMC5623137 28703488

[B15] Gomez-Lopez N. Romero R. Xu Y. Miller D. Unkel R. Shaman M.. (2017b). Neutrophil extracellular traps in the amniotic cavity of women with intra-amniotic infection: a new mechanism of host defense. Reprod. Sci. 24, 1139–1153. doi: 10.1177/1933719116678690 27884950 PMC6343453

[B16] Hassan S. S. Romero R. Maymon E. Berry S. M. Blackwell S. C. Treadwell M. C.. (2001). Does cervical cerclage prevent preterm delivery in patients with a short cervix? Am. J. obstetrics gynecology 184, 1325–1331. doi: 10.1067/mob.2001.115119 11408848

[B17] Hu M. Li H. Li G. Wang Y. Liu J. Zhang M.. (2023). NETs promote ROS production to induce human amniotic epithelial cell apoptosis via ERK1/2 signaling in spontaneous preterm birth. Am. J. Reprod. Immunol. 89, e13656. doi: 10.1111/aji.13656 36409534

[B18] Laudanski P. Lemancewicz A. Kuc P. Charkiewicz K. Ramotowska B. Kretowska M.. (2014). Chemokines profiling of patients with preterm birth. Mediators Inflammation 2014, 1–7. doi: 10.1155/2014/185758 PMC4020160 24876667

[B19] Leaños-Miranda A. Nolasco-Leaños A. G. Carrillo-Juárez R. I. Molina-Pérez C. J. Isordia-Salas I. Ramírez-Valenzuela K. L. (2021). Interleukin-6 in amniotic fluid: A reliable marker for adverse outcomes in women in preterm labor and intact membranes. Fetal Diagnosis Ther. 48, 313–320. doi: 10.1159/000514898 33794521

[B20] Lee S. E. Romero R. Park C.-W. Jun J. K. Yoon B. H. (2008). The frequency and significance of intraamniotic inflammation in patients with cervical insufficiency. Am. J. obstetrics gynecology 198, 633. e631–633. e638. doi: 10.1016/j.ajog.2007.11.047 18342290

[B21] Liu. Y. Liu Y. Zhang R. Zhu L. Feng Z. (2016). Early- or mid-trimester amniocentesis biomarkers for predicting preterm delivery: a meta-analysis. Ann. Med. 49 (1), 1–10. doi: 10.1080/07853890.2016.1211789 27494609

[B22] Manokhina I. Del Gobbo G. F. Konwar C. Wilson S. L. Robinson W. P. (2017). Placental biomarkers for assessing fetal health. Hum. Mol. Genet. 26, R237–R245. doi: 10.1093/hmg/ddx210 28595268

[B23] Martinez-Varea A. Romero R. Xu Y. Miller D. Ahmed A. I. Chaemsaithong P.. (2017). Clinical chorioamnionitis at term VII: the amniotic fluid cellular immune response. J. perinatal Med. 45, 523–538. doi: 10.1515/jpm-2016-0225 PMC5624709 27763883

[B24] Mays J. K. Figueroa R. Shah J. Khakoo H. Kaminsky S. Tejani N. (2000). Amniocentesis for selection before rescue cerclage. Obstetrics Gynecology 95, 652–655. doi: 10.1016/s0029-7844(99)00633-x 10775723

[B25] Mendz G. L. Kaakoush N. O. Quinlivan J. A. (2013). Bacterial aetiological agents of intra-amniotic infections and preterm birth in pregnant women. Front. Cell. infection Microbiol. 3, 58. doi: 10.3389/fcimb.2013.00058 PMC3797391 24137568

[B26] Miao Q. Ma Y. Wang Q. Pan J. Zhang Y. Jin W.. (2018). Microbiological diagnostic performance of metagenomic next-generation sequencing when applied to clinical practice. Clin. Infect. Dis. 67, S231–S240. doi: 10.1093/cid/ciy693 30423048

[B27] Nader E. Romana M. Connes P. (2020). The red blood cell—inflammation vicious circle in sickle cell disease. Front. Immunol. 11, 517556. doi: 10.3389/fimmu.2020.00454 PMC7082402 32231672

[B28] Oh K. J. Lee S. E. Jung H. Kim G. Romero R. Yoon B. H. (2010). Detection of ureaplasmas by the polymerase chain reaction in the amniotic fluid of patients with cervical insufficiency. 38 (2010), 261–268 doi: 10.1515/jpm.2010.040 PMC3085903 20192887

[B29] Onderdonk A. B. Hecht J. L. McElrath T. F. Delaney M. L. Allred E. N. Leviton A. (2008). Colonization of second-trimester placenta parenchyma. Am. J. Obstet Gynecol 199, 52.e51–52.e10. doi: 10.1016/j.ajog.2007.11.068 PMC2827873 18313635

[B30] Romero R. Dey S. K. Fisher S. J. (2014a). Preterm labor: one syndrome, many causes. Science 345, 760–765. doi: 10.1126/science.1251816 25124429 PMC4191866

[B31] Romero R. Gervasi M. T. DiGiulio D. B. Jung E. Suksai M. Miranda J.. (2023). Are bacteria, fungi, and archaea present in the midtrimester amniotic fluid?. J Perinat Med 51(7). doi: 10.1515/jpm-2022-0604 PMC10482702 37194083

[B32] Romero R. Gonzalez R. Sepulveda W. Brandt F. Ramirez M. Sorokin Y.. (1992). Infection and labor: VIII. Microbial invasion of the amniotic cavity in patients with suspected cervical incompetence: prevalence and clinical significance. Am. J. obstetrics gynecology 167, 1086–1091. doi: 10.1016/s0002-9378(12)80043-3 1415396

[B33] Romero R. Miranda J. Chaiworapongsa T. Korzeniewski S. J. Chaemsaithong P. Gotsch F.. (2014b). Prevalence and clinical significance of sterile intra-amniotic inflammation in patients with preterm labor and intact membranes. Am. J. Reprod. Immunol. 72, 458–474. doi: 10.1111/aji.2014.72.issue-5 25078709 PMC4192099

[B34] Schnyder-Candrian S. Walz A. (1997). Neutrophil-activating protein ENA-78 and IL-8 exhibit different patterns of expression in lipopolysaccharide-and cytokine-stimulated human monocytes. J. Immunol. (Baltimore Md: 1950) 158, 3888–3894. doi: 10.4049/jimmunol.158.8.3888 9103458

[B35] Šket T. TŽ R. Starčič Erjavec M. Kreft M. E. (2021). The role of innate immune system in the human amniotic membrane and human amniotic fluid in protection against intra-amniotic infections and inflammation. Front. Immunol. 12, 735324. doi: 10.3389/fimmu.2021.735324 34745106 PMC8566738

[B36] Tadie J.-M. Bae H.-B. Jiang S. Park D. W. Bell C. P. Yang H.. (2013). HMGB1 promotes neutrophil extracellular trap formation through interactions with Toll-like receptor 4. Am. J. Physiology-Lung Cell. Mol. Physiol. 304, L342–L349. doi: 10.1152/ajplung.00151.2012 PMC3602738 23316068

